# “This conflict has turned me into a Doctor, Nurse and Nutritionist at the same time”: how peer support among HIV-positive IDPs created opportunities for care in conflict-stricken Kabaré, Congo DR

**DOI:** 10.1186/s13104-019-4162-y

**Published:** 2019-03-12

**Authors:** Choolwe Muzyamba

**Affiliations:** 1 Maastricht Graduate School of Governance/UNU-Merit, Maastricht University, Maastricht, The Netherlands; 2 A9 Marshlands, Village Box 32379, Lusaka, Zambia

**Keywords:** HIV/AIDS, Congo DR, Internally-displaced-persons

## Abstract

**Objective:**

While HIV research remains priority in Sub-Saharan Africa (SSA), most of the studies have traditionally been conducted in secure locations with little focus on internally displaced person (IDPs) and how they rely on locally available strategies for care and survival. Thus the aim of this study is to fill this gap by investigating the role of indigenous social relations (particularly, peer support) in the promotion of care among IDPs living with human immunodeficiency virus (HIV) in a conflict region known as Kabaré in the south Kivu province of Eastern Democratic Republic of the Congo.

**Results:**

Through a qualitative study, we show that despite having some limitations (e.g. lacked practical avenues to monitor and treat HIV-related complications), peer-support was crucial in providing much needed empathetic social, economic, psychological, material, nutritional and emotional supportive services to HIV positive IDPs. Peer support was also useful in promoting adherence to antiretroviral treatment including provision of financial support that opened survival pathways in the face of conflict, weak health systems and poverty.

## Introduction

While research on Human Immunodeficiency Virus (HIV) remains priority within public health in Sub-Saharan Africa (SSA), most of the studies have traditionally been conducted in secure locations with specific focus on formal interventions (by governments, Non-Governmental Organizations-NGOs, or donors) [1]. Areas of conflict have generally been neglected [2, 3]. This is despite the fact that HIV-infected people living in conflict regions face an even greater challenge given the constant threat to their lives from armed conflict [2, 4]. Further, the HIV response has mainly focused on formal responses with little focus on the role played by community support systems despite them being the most widely used by people on the ground given the weak health system in conflict regions [5, 6]. In particular, indigenous social relations that help promote opportunities for care and support among internally displaced people (IDPs) with HIV [7, 8]. Research has shown that in SSA, formal interventions only carry a small proportion of the burden; the bulk of the burden is carried by the communities in which the HIV-infected live their daily lives [9, 10]. However, little is known about how the combination of conflict and HIV prevalence creates indigenous opportunities of care and support for IDPs with HIV [2, 7, 8]. This study aims to contribute to filling this gap through a case study on IDPs living with HIV in Kabaré city, South Kivu province of Congo DR. Specifically, it aims to investigate the role of indigenous social relations (particularly, peer support) in the promotion of care among IDPs with HIV in a conflict region known as Kabaré in the south Kivu province of Eastern Democratic Republic of the Congo. Within HIV response, Peer support is conceptualized as assistance by one community member to another [6]. It usually takes the form of financial, emotional, logistical, psychological, and material assistance [8, 11]. In extant literature, peer support has been associated with increased capacity to identify and respond to HIV-related problems in context-specific ways [1, 5, 12].

### Theoretical framework

The study makes use of the social psychological framework known as “community health competence” which was advanced by Campbell and Cornish [1]. The framework emphasizes the role that informal community support systems such as peer-support play in producing opportunities of care for people with HIV in resource-poor settings [1, 12]. The framework holds that support from and between community members facilitates or hinders the development of heath-enhancing environments more sustainably [1]. Thus the “Community health competence” is a conceptual framework which enables researchers to assess how community resources (such as peer-support) produce (or fail to produce) health-enhancing environments for local people by relying on “local culture, context and local survival strategies” and collaboration with external expert [1]. In this study, the framework is used to structure the analysis of how peer support either facilitates or hinders promotion of care among HIV positive IDPs in Kabaré [9, 10].

## Main text

### Methods

#### Ethical approval

We obtained written ethical clearance from Comité d’Ethique du Nord-Kivu (Ethics Committee of North-Kivu)—Université Catholique du Graben (Catholic University of Graben). Other than that, during focus group discussions (FGDs), we collected written informed consent from the participants before participation, and at the same time, participants were made aware of their right to discontinue their participation at any point should they wish to.

#### Setting

Our study was conducted in Kabaré which is an administrative district of the South Kivu Province in eastern Democratic Republic of the Congo. According to the UNAIDS (2016), armed conflict has persisted in this region since 1998 making it the most insecure and hungriest region in Africa [4]. In this region, people continue to lack basic services such as food, decent shelter and health services [4]. HIV is also prevalent in this region with around 3% of the population living with HIV [4].

#### Sampling method

We relied on purposive sampling technique by establishing contact with two local community groups that are working with HIV positive IDPs. Our participants included IDPs living with HIV who had expressed interest to participate in the study. We ensured diversity in participants in terms of age, level of education, gender and ethnicity. This was done in order to increase the diversity of opinions expressed and to allow for varied discussions.

#### Data collection method

As mentioned above, qualitative data were collected in Kabaré in the eastern Democratic Republic of the Congo in 2017. The data collection process consisted of 4 FGDs all involving HIV-positive IDPs who had expressed interest in participating. Each of the FGDs had 10 participants and the last one had 8 participants bringing our total number of participants to 38. In line with the framework explained above, we structured topic guides that endeavored to elucidate how peer support either promoted or hindered opportunities of care for HIV positive IDPs. This was done through asking questions that allowed participants to explain the different ways they experienced peer support, how useful they found it vis-à-vis HIV care, including the challenges that were associated with Peer support as tool for promoting care for them. We also ensured that follow-up questions were asked for clarification purposes, and to also encourage respondents to expand on some of their answers. The FDGs lasted an average of 60 to 90 min, and they were held in a secure room at the social center of Kabaré which is managed by community leaders. The FGDs were conducted in the local language of Lingala. The data were digitally recorded and later transcribed and translated to English.

#### Analysis

After transcribing and translating the transcripts, we used NVivo to conduct thematic analysis [13]. The “community health competence” framework directed our analysis by ensuring that we focused on the varied ways peer support either promoted or hindered care for HIV-positive IDPs. This was done by clustering our data into basic themes and later into organizing and global themes thereby clearly demonstrating the varied ways peer support either promoted or hindered care for HIV positive IDPs (see Table 1).

**Table 1:** Summary of results

Global theme	Organizing theme	Basic theme identified in the FGDs
Usefulness of peer-support	Promoted dialogue on HIV risk and prevention	Allowed for dialogue on safe sex
		Allowed for discussion on PMTCT
		Allowed for information on treatment adherence
	Provided different forms of support to IDPs	Peer support promoted emotional, psychological, physical and economic support among HIV positive people
	Base for fighting disenfranchisement, marginalization, stigma and discrimination	Allowed IDPs to collectively challenge disenfranchisement, marginalization stigma and discrimination Develop agency to fight for improved care
	Utilization of indigenous resources in absence of efficient health systems	Provide continuous home-based maternal care
		Provided each other with locally-available nutritious meals through group cooks (Mungaano) e.g. soya beans, coffee etc.
	Promoted treatment-adherence	Peer support encouraged regular and consistent uptake of ARVs before
		Peer support served as a continuous reminder for uptake of ARVs
	Fostered alliances	Peer support allows for the formation of alliances among peers to advocate for an end to sexual cleansing
		Fighting patriarchy and promoting women empowerment
	As a conduit for referrals to professional care	Helped provide safe-passage to health facilities
		Help to provide transportation support to health facilities
Peer support limitations	Peers lacked technical skills, equipment and medical supplies to handle complications	Peer lack skills to Help Easily conduct HIV tests
		Peers lack skills and equipment to monitor CD4 count
	Reinforced a stereotypes about ARVS	People branding ARVs as a neocolonial project aimed at eliminating them

### Results

Our results show that although it has some limitations, peer-support is largely characterized as a force for good (See Table 1). Particularly, in an effort to fight different challenges faced by IDPs with HIV in conflict-stricken Kabaré. It was clear that peer-support provided safe spaces for dialogue amongst peers, which enabled sharing of helpful information specific to IDPs with HIV. This information included the importance of adherence to treatment, safe-sex and Prevention of Mother to Child Transmission (PMTCT). Further, in a place like Kabaré where people are internally displaced, poverty was rife and health systems dysfunctional; thus peer support acted as an avenue for providing financial, emotional, psychological, physical and material support to IDPs living with HIV.

*“For me, I have seen that my colleagues are supportive to each other through different forms. I have personally benefited emotionally, psychologically and also physically because my colleagues where present to help me”* participant 11

Among HIV positive IDPs, peer support was also seen as a means for fighting disenfranchisement, marginalization, stigma and discrimination resulting from their HIV status. It was a means for building solidarity in an effort to deconstruct and challenge different forms of oppression precipitated by their HIV status within the conflict-stricken community. According to our participants, peer support allowed them to develop agency (the motivation and capacity) to foster improved care for themselves and also to assert their needs in relation to their HIV-vulnerability as IDPs.

*“we now work in unit to challenge the bad treatment, stigma and discrimination which we have been subjected to for a long time. We now speak as one voice”* participant 3

Further, challenges resulting from being poor, displaced, and HIV positive were tackled by drawing on indigenous resources. IDPs were provided with home-based care and nutritious. In this way, IDPs with HIV were able to access necessary nutrition and care which they would otherwise not have accessed given the constrains in this community.

*“We grow crops and share to enhance our health. Soya beans is important for us with HIV”* participant 22

Peer support was also useful in fostering alliances necessary for challenging patriarchy and sexual cleansing which were rampant in the communities. These alliances also served as a conduit for referrals to health facilities in order to access regular professional check-ups and care.

*“You can see how colleagues have been effective in working together to fight patriarchy, sexual cleansing and at the same time, we also help each other to go to the clinic”* participant 10

However, despite peer support generally being characterized as a force for good, IDPs living with HIV highlighted a number of limitations associated with peer support within their context. For example, they were critical of how peer support was only a symbolic tool which lacked practical and technical means to monitor and treat HIV. They highlighted how most community members lacked technical skills, equipment and medical supplies to handle several complications that arise due to their HIV status. They were also critical of how peer support sometimes reinforced negative stereotypes about HIV treatment. Particularly, they complained about how some community members were skeptical of antiretroviral therapy suggesting that it could be “a neocolonial strategy by the West to eliminate them”. This affected adherence to treatment among some IDPs with HIV.

*“we get several complications and this peer support is powerless. What can my friends do to treat my kaposi’s sarcoma? We do not have necessary skills, medication and equipment to handle deeper challenges”* participant 21

### Discussion

Based on the community health competence framework, our findings show the varied ways peer support promotes and hinders care for HIV positive IDPs. In the case of Kabaré, the last two decades of conflict have led to the collapse of health systems and stifled access to food, work and any other means of survival for residents [4]. IDPs with HIV in this region face even greater challenges given that their survival is threatened by armed conflict, stigma and discrimination and lack of professional care [2, 4]. Given all these challenges, peer-support was crucial in providing much needed economic, psychological, material, nutritional and emotional supportive services to HIV positive IDPs. This is in line with other studies within SSA suggesting that peer support is a useful complement to weak health systems within HIV response [5, 11].

Peer support was also useful in promoting adherence to antiretroviral treatment including provision of financial support that opened survival pathways in the face of poverty. While it is true that in general peer support is a force for good, it does however present some limitations. For example, it was sometimes used to reinforce negative attitudes towards antiretroviral therapy which negatively affected adherence to treatment. Further, peers lacked skills, equipment and drugs to handle most possible complications among IDPs living with HIV. While peer support opens up different opportunities of survival, it is not a replacement for formal care. It can be seen as a useful compliment to formal care in areas of conflict where professional care is either absent or inadequate [9]. Thus despite its limitations, it is to many IDPs the only feasible avenue of care. Therefore, incorporating peer support in HIV response allows for the identification of its shortcomings and also provides an opportunity to address them. At the same time, incorporating peer support also allows for the enhancement of its potential benefits.

Further, our findings are consisted with other studies [3, 5, 6, 8, 11, 14] elsewhere in which peer support is praised for providing opportunities for enhanced well-being amongst extremely vulnerable groups. This speaks to the need for greater attention to local efforts which are often regarded as secondary to formal interventions by health professionals [1]. In conflict regions where these formal responses are either absent or inadequate, peer support opens up possibilities for tackling several challenges in context-specific ways [2–4]. In agreement with other studies on peer support, we come to the conclusion that partnership-building among IDPs with HIV and enhancing their ‘voice’ helps improve their agency and allows them to contextually-respond to their challenges in ways that are feasible. It seems there is more to benefit from recognizing specific local needs and enhancing networks of survival, and align HIV care policies in a manner that maximizes opportunities of care in conflict regions. An uncritical direct top-down implementation of policies might obscure such networks which have been used as avenues of care and survival in conflict regions.

In sum, the study with the help of the community health competence framework shows that IDPs living with HIV in Kabaré face intersecting challenges; these include stigma and discrimination, weak or lack of proper health systems and threat of armed conflict. This makes their access to care very difficult. However, peer support has provided opportunities for care. In this sense, peer-support is crucial in providing much needed social, economic, psychological, material, nutritional and emotional supportive services to HIV positive IDPs. That notwithstanding, peer support has some limitations; particularly, it can be used to reinforce negative attitudes towards antiretroviral treatment and in some ways, most peers lack HIV technical skills and equipment. Despite these limitations, to many IDPs, peer support remains the only available and feasible avenue of care. Thus peer support should be included in HIV response either to promote its potential benefits or limit its costs.

## Limitations

Firstly, against a backdrop of widespread conflict, our findings are based only on the views of HIV positive IDPs residing in a single region of Congo DR. This fact limits the variety of experiences with peer support in Congo DR in general. However, we argue that this study provides useful insights and the first step in understanding the different ways peer support hinders/promotes care among HIV positive IDPs in conflict regions.

## Abbreviations

FGD: focused group discussion
HIV: human immunodeficiency virus
IDPs: internally displaced persons
NGO: Non-Governmental Organization
SSA: Sub-Saharan Africa
WHO: World Health Organization

## References

[1] Campbell C, Cornish F (2012). How can community health programmes build enabling environments for transformative communication? Experiences from India and South Africa. AIDS Behav.

[2] UNHCR (2006). HIV/AIDS and internally displaced persons.

[3] Olakunde BO, Mamadu I, Olaifa Y, Wakdok S (2016). Internally displaced persons (IDP) and HIV in Nigeria: Outcome of HCT outreach to IDP camps in Northeast Nigeria. J AIDS Clin Res..

[4] USAID (2016). Country specific information: Democratic Republic of Congo (DRC) multi-year development food assistance projects fiscal years 2016–2020.

[5] Simoni J, Nelson K, Franks J (2011). Are peer interventions for HIV efficacious? A systematic review?. AIDS Behav.

[6] Cabral H, Davis-Plourde K, Sarango M, Fox J, Palmisano J, Rajabiun S (2018). Peer support and the HIV continuum of care: results from a multi-site randomized clinical trial in three urban clinics in the United States. AIDS Behav.

[7] Campbell C, MacPhail C (2002). Peer education, gender and the development of critical consciousness: participatory HIV prevention by South African youth. Soc Sci Med.

[8] Muzyamba C, Groot W, Pavlova M, Tomini S (2018). Community mobilization and maternal care of women living with HIV in poor settings: the case of Mfuwe, Zambia. BMC Health Serv Res..

[9] Campbell C, Cornish F (2010). Towards a ‘‘fourth generation’’ of approaches to HIV/AIDS management: creating contexts for effective community mobilisation. AIDS Care.

[10] Nhamo M, Campbell C, Gregson S (2010). bstacles to local-level AIDS competence in rural Zimbabwe: putting HIV prevention in context. AIDS Care.

[11] Muzyamba C, Groot W, Tomini S, Pavlova M (2017). The role of community mobilization in maternal care provision for women in sub-Saharan Africa—a systematic review of studies using an experimental design. BMC Pregnancy Childbirth..

[12] Campbell C, Coultas C, Andersen L, Broaddus E, Skovdal M, Nyamukapa C, Gregson S (2015). Conceptualising schools as a source of social capital for HIV affected children in southern Africa. LSE Res Online..

[13] Braun V, Clarke V (2006). Using thematic analysis in psychology. Qual Res Psychol.

[14] Dutcher M, Phicil S, Goldenkranz S (2011). Positive examples’’: a bottom-up approach to identifying best practices in HIV care and treatment based on the experiences of peer educators. AIDS Patient Care STDs.

